# New Appliances and Things Medical

**Published:** 1906-06-02

**Authors:** 


### NEW APPLIANCES AND THINGS MEDICAL.

[We shall be glad to receive at our Office, 28 & 29 Southampton Street, Strand, London, W.C., from the manufacturers, specimens of all new preparations and appliances which may be brought out from time to time.]

### WORLDS' ANTISEPTIC AMMONIA.

(Worlds and Company, Seymour House, 17 Waterloo Place, London, S.W.)

We have made an extensive trial of this antiseptic preparation of ammonia and find that it is in every way to be recommended for the purposes indicated upon the bottle. When added to the extent of two tablespoonfuls to an ordinary bath it has a marked effect in softening the water and at the same time exerts a stimulant action upon the vessels of the skin, the latter giving rise reflexly to cardiac and respiratory stimulation. It is also exceedingly useful as an application to insect bites, and travellers in countries infested with gnats and mosquitos will find Worlds' antiseptic ammonia an exceedingly valuable adjunct to their kit. In addition to its value for the above medical purposes, it seems to have an extensive sphere of usefulness for domestic objects such as the cleansing of linen, plate, hairbrushes, etc. We are quite sure that this form of antiseptic ammonia will supply a conside[illegible]le need and be greatly appreciated both by the public and the profession.

